# Beyond Size and Class Balance: Alpha as a New Dataset Quality Metric for Deep Learning

**DOI:** 10.3390/bioengineering13080854

**Published:** 2026-07-24

**Authors:** Josiah D. Couch, Rima Arnaout, Ramy Arnaout

**Affiliations:** 1 Department of Pathology, Beth Israel Deaconess Medical Center (BIDMC), Boston, MA 02215, USA; 2 Department of Medicine, University of California San Francisco, San Francisco, CA 94143, USA; 3 Bakar Institute for Computational Health Sciences, University of California San Francisco, San Francisco, CA 94143, USA; 4 Center for Intelligent Imaging, University of California San Francisco, San Francisco, CA 94143, USA; 5 Division of Clinical Informatics, Department of Medicine, Beth Israel Deaconess Medical Center (BIDMC), Boston, MA 02215, USA; 6 Harvard Medical School, Harvard University, Boston, MA 02115, USA

**Keywords:** computer vision, artificial neural networks, entropy, diversity, similarity, dataset quality, computational pathology, computational cardiology, medical imaging

## Abstract

In deep-learning-based image classification, achieving high performance requires diverse training sets. However, the current best practice—maximizing dataset size and class balance—does not guarantee dataset diversity. We hypothesized that, for a given model architecture, performance improves by maximizing diversity more directly. To test this hypothesis, we introduce a comprehensive framework of “sentropic” diversity measures or “sentropies” from ecology that generalizes familiar quantities like Shannon entropy by accounting for similarities among images. Size and class balance emerge as special cases. Analyzing thousands of subsets from seven medical-imaging datasets showed that the best correlates of performance were not size or class balance but *A*—“big alpha”—a set of sentropies interpreted as the effective number of image–class pairs in the dataset, after accounting for similarities among images. One of these, $A_{0}$, explained 67% of the variance in balanced accuracy, vs. 54% for class balance and just 39% for size. The best pair of measures was size-plus-$A_{1}$ (79%), which outperformed size-plus-class-balance (74%). Subsets with the largest $A_{0}$ performed up to 16% better than those with the largest size (median improvement, 8%). We propose developing methods to maximize *A* as a way to improve deep learning performance in medical imaging.

## 1. Introduction

For deep learning to reach its potential in medical imaging, models must be data-efficient, highly performant, and generalizable [1,2,3]. Investigators have long appreciated that models generally perform better on datasets that are more diverse. As a working definition, a diverse dataset is one whose elements are sufficiently representative of the intended real-world application for a model to be able to learn class invariants that generalize beyond the dataset. Perhaps the most commonly used strategy to promote diversity is to maximize dataset size and class balance: the larger the dataset, the more likely its elements will fill out the space of real-world possibilities; the more class-balanced, the more elements per class.

Indeed, size and class balance have long been the two primary heuristics of dataset quality, which investigators have sought to maximize for applications including (but not limited to) image classification, the focus of the present work, since well before the explosion of deep learning over the past 20 years [4,5,6,7,8,9].

Yet from this working definition, it should be clear that size and class balance alone will not generally provide a complete description of dataset diversity, since they only indicate how many images there are, overall and in each class; they do not indicate how well these elements fill out the space of possibilities, which is related to how similar or different the images in the dataset or class are to each other. Given that constructing large, class-balanced datasets is often challenging—especially in medicine, where acquiring and annotating high-quality images can be expensive and/or labor-intensive—the conventional approach of seeking to maximize size and class balance might not always be the most efficient strategy for achieving high-quality datasets. To improve on current practice, one approach is to ask whether there exist measures of dataset diversity that account for similarities and differences among the elements in the dataset, and if so, whether these measures might outperform size and class balance as correlates of model performance (for a given model architecture).

The present work demonstrates that the answer to both questions is “yes,” suggesting a new potential avenue for improving machine learning performance by optimizing measures of dataset quality that capture similarities and differences among dataset elements. Specifically, we identify *A*, “big alpha,” and more specifically measures known as $A_{q\;=\;0}$ (“big alpha at *q* equals 0;” henceforth $A_{0}$) and $A_{1}$, as the best correlates of AUC, F1 score, accuracy, and balanced accuracy, using ResNet-18 and a variety of medical-imaging datasets across a range of imaging modalities as proof of principle. *A* is part of a comprehensive mathematical framework developed in ecology over the past decade that generalizes the Rényi entropies [10] (of which Shannon entropy [11] is a special case) to create a family of similarity-sensitive diversity measures. We refer to this framework as “sentropy,” for similarity-sensitive entropy, and the individual measures within this framework as sentropies (Section 2). Using ultrasound, X-ray, computed tomography (CT), histopathology, and hematology image datasets, we present an approach for assessing how well each diversity measure correlates with performance (Section 3), and describe the results of this approach (Section 4). We conclude with a discussion of limitations and future directions (Section 5).

## 2. Relationship to Prior Work

There is a rich literature describing issues related to dataset composition in the context of machine learning, and an extensive literature on potential solutions. Data augmentation is a standard solution in imaging (and other) applications that was originally introduced as a way to increase dataset size, although even early works make it clear that the implicit goal was to increase diversity (an early example of augmenting size as a proxy for increasing diversity) [4,5,6,7,9]. The value of class balance as a contributor to model performance has long been recognized and has been extensively described. Regarding evaluation of model performance, prior work has also described the danger of using “vanilla” accuracy for performance evaluation when test sets are imbalanced [12], since in cases of severe imbalance a model may be able to achieve high accuracy simply by ignoring the smallest class(es); the general solution has been to evaluate performance using balanced accuracy (BACC) instead (related to the F1 score), as we do in the present work.

Investigations of dataset “complexity,” by various definitions, as a determinant of model performance independent of size and class balance predate discovery of sentropy by over a decade [13]. This includes appreciation of within-class diversity, discussed in the context of within-class imbalance and the problem of small disjuncts [14,15,16]. Such work demonstrates long-standing appreciation of a world beyond size and class balance, although of course not investigated systematically according to sentropy or to our knowledge in the context of modern deep learning or medical imaging. More generally, many methods for measuring dataset complexity have been proposed, some defining complexity specifically in the context of model performance (e.g., “hardness”) and others defining it based on dataset and/or image composition, but none using sentropy [17,18,19,20]. Of note, some of the latter methods employ entropy within images (e.g., color entropy [21]) as opposed to across images within a dataset, as we do here; the focus of this prior work was investigating how best to estimate similarities between images, an important issue that is largely beyond the scope of the present work, as opposed to measuring the diversity of the dataset as a whole, which is our focus in the present work.

Finally, data optimization methods [22] such as dataset pruning, distillation, and boosting [23,24,25,26]; instance selection [27]; and to some extent active learning [28,29] are all concerned with creating smaller datasets that achieve the same model performance as larger ones. In this sense, all are motivated by the goal of preserving dataset diversity (indirectly and/or by other names) irrespective of size and in some cases of class balance, by maximizing bespoke quantities whose potential relationships to the first-principles entropic formulation of sentropy as a unifying framework are as yet uninvestigated. However, the emphasis in these prior works was how to optimize these quantities (which we speculate might prove to be correlates of sentropy in some fashion), whereas the goal of the present work is to determine what specific sentropic measure(s) best predict model performance (with the question of how best to optimize them, algorithmically or mechanistically, left for future investigation).

In addition to these optimization frameworks, predicting model performance from a set of features, especially training set size, has been a major topic in the neural scaling laws literature, both empirically [30,31,32,33] and theoretically [34,35,36,37]. This has specifically intersected the pruning literature in work that has suggested that performance can be improved by keeping either the “hardest” or “easiest” training examples [38,39]. However, this work was not cast in information-theoretic terms such as (s)entropy.

**Figure 1:**
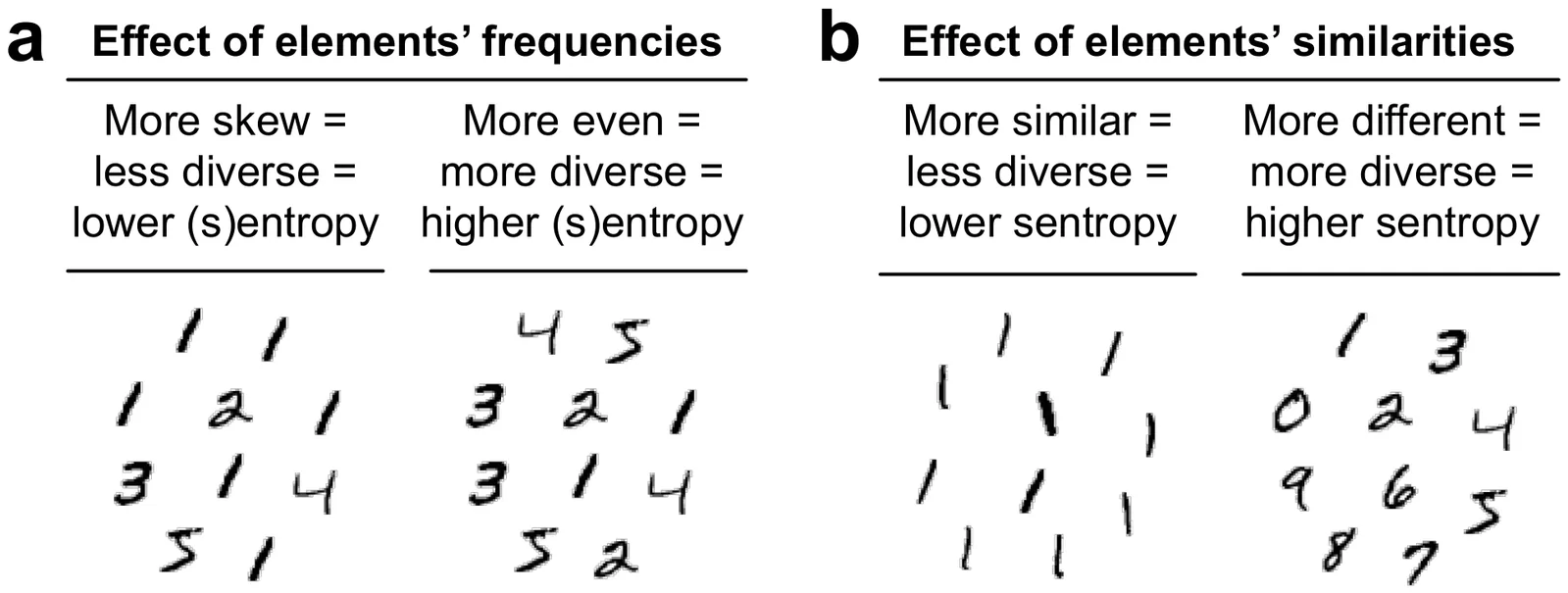
Sensible definitions of diversity must be sensitive to both frequency and similarity. Four same-sized datasets of 10 images each from the MNIST handwritten digits dataset [47] are shown. The two datasets in (**a**) contain the same five unique images, differing only in their relative frequencies; the more balanced dataset is intuitively more diverse. The two datasets in (**b**) each contain 10 unique images but differ in how similar the images are to each other; the dataset with the more-different images is intuitively more diverse. See also, e.g., [48,49].

**Figure 2:**
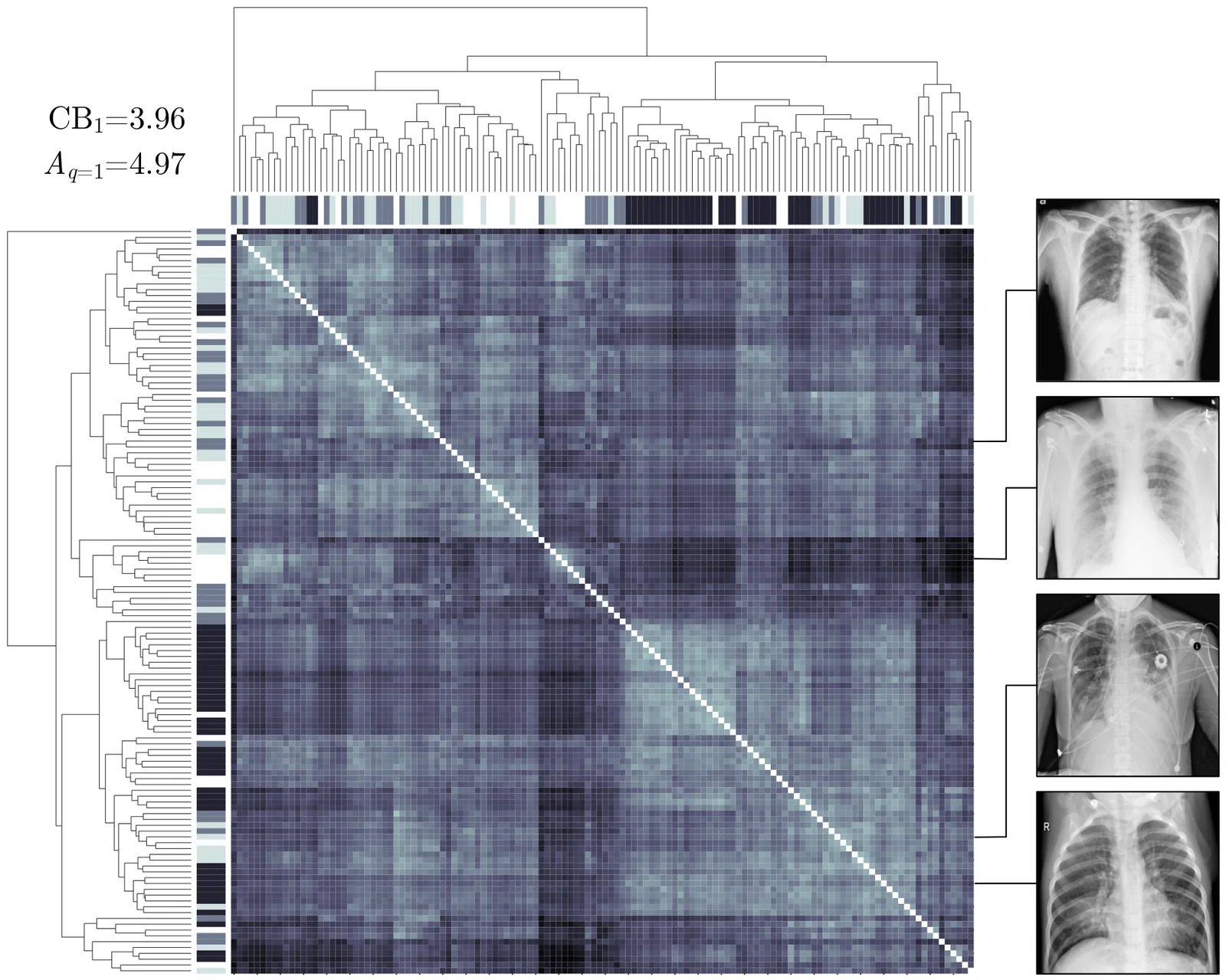
Interpreting the diversity of a dataset by visualizing soft clusters within the similarity matrix. Right: four randomly chosen images from different regions of the clustered heatmap, one from each of the four classes.

We use the term “sentropy” as a portmanteau for similarity-sensitive entropy, a concept introduced in a framework developed by Leinster, Cobbold, and Reeve [40,41]. An overview and intuitive interpretation are presented in Appendix A.1, Appendix A.2, Appendix A.3, Appendix A.4, Appendix A.5 and Appendix A.6; see [42] for details. The Vendi score family of measures [43,44,45] represents a different but similar sentropic framework. Sentropy argues that simply considering the size and frequency of elements within a dataset is insufficient for reasonable measures of diversity; similarity between those elements is also crucial (Figure 1). The framework builds upon Hill’s D-number system [46], which measures diversity based on element frequencies; sentropy extends Hill by incorporating a similarity matrix to account for relationships between elements. This enables the calculation of within- and between-class sentropies, analogous to relative entropy (Kullback–Leibler divergence), cross entropy, and mutual information. Sentropy effectively allows for “soft clustering” based on element similarities, capturing overlap and nuance not possible with traditional methods (Figure 2). We apply these measures to dataset diversity, as possible correlates with the performance of deep learning models, going beyond simple frequency counts to capture the true complexity of the data.

The Leinster–Cobbold–Reeve and Vendi frameworks have been compared in the context of machine learning datasets [50], but not as correlates of model performance. Vendi scores always bound the analogous LCR measure; LCR is cheaper to compute, rationalizing our focus on LCR here.

## 3. Materials and Methods

### 3.1. Selecting Datasets

Datasets were selected to represent diverse imaging modalities, provided the following criteria were met: the dataset was large enough to allow extensive subsampling (≥10,000 images), the images were labeled mutually exclusively, and published benchmark accuracy was sufficiently high (≥90% as reported in the relevant literature).

MedMNIST is a public collection of datasets of different imaging modalities [51,52]. Four MedMNIST datasets met the above criteria: BloodMNIST, PathMNIST, OrganAMNIST, and OrganCMNIST. BloodMNIST contains resized, center-cropped, color light-microscope images of single cells from peripheral blood smears from healthy human individuals, with eight classes labeled by cell type: basophil, eosinophil, erythroblast, immature granulocyte, lymphocyte, monocyte, neutrophil, and platelet. PathMNIST is a set of light-microscope images of non-overlapping image patches from hematoxylin–eosin-stained histological slides from colorectal cancer cases, with nine classes labeled by tissue type: tumor epithelium, simple stroma, complex stroma, mucosal lymphoid follicle, adipose tissue, debris, normal mucosal glands, duct and vessels, and muscle. OrganAMNIST and OrganCMNIST are, respectively, axial- and coronal-cut computed tomography (CT) images from the Liver Tumor Segmentation Benchmark (LiTS) [53] with 11 classes labeled by organ or bone: bladder, femur-left, femur-right, heart, kidney-left, kidney-right, liver, lung-left, lung-right, pancreas, and spleen.

Three non-MedMNIST datasets were also used, which we refer to as CHD, AVC, and COVID. The CHD dataset consists of fetal echocardiogram images (ultrasounds) from [54] labeled by view into three classes: apical four-chamber (A4C), three-vessel (3VV), and nontarget (NT). In ultrasound, one or more clips are acquired per patient; each clip consists of one to several hundred consecutive image frames. Training and test sets were divided by patient identifier and were disjoint from each other [27]. AVC is a dataset of echocardiographic images from 267 different studies from adult patients [55], labeled into 15 classes by view: apical 2 chamber (A2C), apical 3 chamber (A3C), A4C, apical 5 chamber (A5C), parasternal long axis (PSLA), short axis basal (SAXBASAL), short axis mid (SAXMID), subcostal 4 chamber (SUB4C), subcostal IVC (IVC), subcostal aorta (SUBAO), right ventricular inflow (RVinflow), and suprasternal aorta (SUPAO). Finally, COVID is the COVID-19 radiography dataset, which consists of chest X-ray images with four classes labeled by diagnosis: normal, non-COVID lung opacity, COVID-19, and viral pneumonia (presumably not from COVID-19) [56,57].

The numbers, sizes, and color depth of the images in each dataset are in Table 1.

### 3.2. Creating Dataset Subsets

Subsets of different sizes and class balances were created for each of the above datasets, as follows. For each subset size *n*, a large number of partitions of *n* into a sum of *m* positive integers was randomly generated, where *m* is the number of classes in the dataset. These were filtered to ensure that the *i*th element of the partition was smaller than the number of images in the *i*th class of the parent training set. For each partition, a corresponding probability distribution was computed by dividing by *n*, and class balance (CB) was measured as the $D_{1}$ of that probability distribution. Partitions were binned into 25 $D_{1}$ bins. For n= 64, 128, 256, 384, 512, 768, or 1024 images, four partitions were chosen at random; for bins with fewer than four partitions, all partitions were selected. For each chosen partition $\left(n_{1},n_{2},\ldots ,n_{m}\right)$, $n_{i}$ images from class *i* were chosen uniformly at random from the parent training set. These images formed a subset. This was repeated for each of the datasets.

### 3.3. Model Training and Performance/Quality Assessment

To ensure a range of scores (by avoiding too-expressive an architecture), a ResNet-18 model [58] was trained on each full dataset (to validate the benchmark performances reported in the literature) and on each subset, using PyTorch 2.1.2 with CUDA 1.21 [59]. The first and last layer were modified to accommodate the differences between datasets in terms of images size and number of classes; the architecture was otherwise unaltered. Because the goal was to study the effect of dataset composition in isolation, no data augmentation was used.

Each model was trained to a training accuracy of ≥99% or for 1000 epochs, whichever came first. The trained models were then tested against a test set common to all the subsets from the given parent dataset. In all cases, the test set was disjoint from the parent dataset and all subsets. The quality of the subset (or of the parent dataset) was measured as the performance of the ResNet: the higher the model performance, the higher the quality of the subset (or parent dataset) was considered to be. Performance was measured using the accuracy (ACC), balanced accuracy (BACC), and multiclass area under the receiver-operator characteristic curve (multiclass AUC), all as implemented in PyTorch. Note that in the PyTorch implementation, AUC is the same as multiclass AUROC under default behavior (micro-averaging); ACC is the same as the F1 score, recall, or precision, again with default behavior (micro-averaging); and BACC is the same as recall with macro-averaging.

### 3.4. Definition of Pairwise Image Similarity and Measurement of Diversity Features

For each of the selected subsets, sentropic diversity features were measured sentropically using the sentropy Python package [49]. The pairwise similarity $Z_{ij}$ between images *i* and *j* was $Z_{ij}=e^{-{\mathrm{RMSD}}_{ij}}$, where RMSD is the pixel-wise root-mean-squared difference.

### 3.5. Quality Indicators/Regression Features

Linear regression was used to measure the adjusted $R^{2}$ between performance and (the logs of) different feature sets: i.e., size, class balance, and different sentropies (by LCR, calculated using the sentropy Python package). Each feature is a potential indicator of dataset quality. A total of 30 features were considered. These included 27 diversity-related features: *A*, *R*, and *G* at each of q=0, 1, and *∞*; minimum α, ρ, and γ at q=0 and 1; maximum α, ρ, and γ at each of q=0, 1, and *∞*; class balance at q=1 and *∞*; and dataset size. (Recall that α, ρ, and γ are per-class sentropies; note that minima of α, ρ, and γ measures at q=∞ are the same as/redundant with *A*, *R*, and *G* at q=∞.) These features were chosen as a manageable number of features that nonetheless span the qualitative and quantitative range of information that sentropy provides. Note that min and max ρ are just the reciprocals of max and min β.

The remaining three features were systematic parameters that differ by dataset but are independent of diversity: image size (the no. of pixels per image), color depth (1 for grayscale images and 3 for color images), and the number of classes (3–15; Table 1). These features were included as a default in all investigations to allow a linear regressor (see next section) the option of using them as normalizing factors. Using the logs allowed the linear regressor to discover scaling relationships, such as class balance scaled by the number of classes (This is because logging converts ratios into differences, with linear coefficients for each feature).

### 3.6. Assessment of Quality Indicators/Features

Regressions were performed on each sentropic feature, each possible pair of sentropic features, each possible set of three sentropic features, and other sets of interest. For robustness, the first two were also performed on each dataset in isolation. The three systematic parameters above were included in every regression, to allow the regression model to normalize by these features. All regressions were performed using the scikit-learn Python package [60]. Adjusted $R^{2}$ was used in order to account for and correct possible overfitting.

### 3.7. Computational Requirements

All computations were done on a single Linux machine with 125GB RAM, an 11th-generation Intel Core i9-11900K CPU running at 3.50 GHz, and an NVIDIA GA102GL RTX A5000 GPU running at 33 MHz. Total runtime to recapitulate the results described in this work on this system was approximately 1 week.

## 4. Results

### 4.1. Overall Goal and Strategy

The goal of this work was to test how dataset quality affects model performance, with quality measured sentropically and performance measured by BACC and related statistics. This was done in three steps. (1) First, for each of several medical imaging datasets, we created many hundreds of image subsets that differed by size, class balance, and diversity. (2) Second, we trained classifiers on each subset using the same reference model architecture (ResNet-18). (3) And third, we regressed performance against sentropic measures—sentropies—including size and class balance as special cases, to determine how well each measure correlates with model performance. In this way, we sought to identify the best correlates of performance from among the measures tested, and to compare them to the current deep-learning benchmarks of size and class balance.

### 4.2. Identifying and Validating High-Performance Datasets

We identified seven large, well characterized, high-quality datasets suitable for analysis (Table 1). To ensure that results would generalize beyond specific cases, these were chosen to cover a rich variety of tissues and organs and a wide range of imaging modalities: cancer histopathology (light microscopy), blood smears (light microscopy), chest radiographs (X-rays), abdominal CTs, and fetal and adult echocardiograms (ultrasound). These datasets included both color and grayscale images and also varied by image size, supporting the generality of the results.

We confirmed the datasets were high quality by requiring excellent performance on classification tasks to have been documented in the literature by AUC, ACC, and/or BACC. We validated these results by training a reference-architecture (ResNet-18) model on each dataset and confirming strong performance, with median AUC, ACC, and BACC of 0.99, 0.92, and 0.88, respectively despite no data augmentation (Table 2). Results from the literature were observed to be slightly better on balance, which was expected because published studies generally used highly specialized and/or fine-tuned models, as opposed to our reference model (whose architecture was held constant except for input and output layers [see Section 3] so that the only variable was dataset quality), as well as possibly due to data augmentation. Performance was sufficiently good to ensure a range of performance values to regress against sentropies, once datasets were divided into subsets.

### 4.3. Model Performance Across Subsets of Varying Size, Class Balance, and Diversity

**Figure 3:**
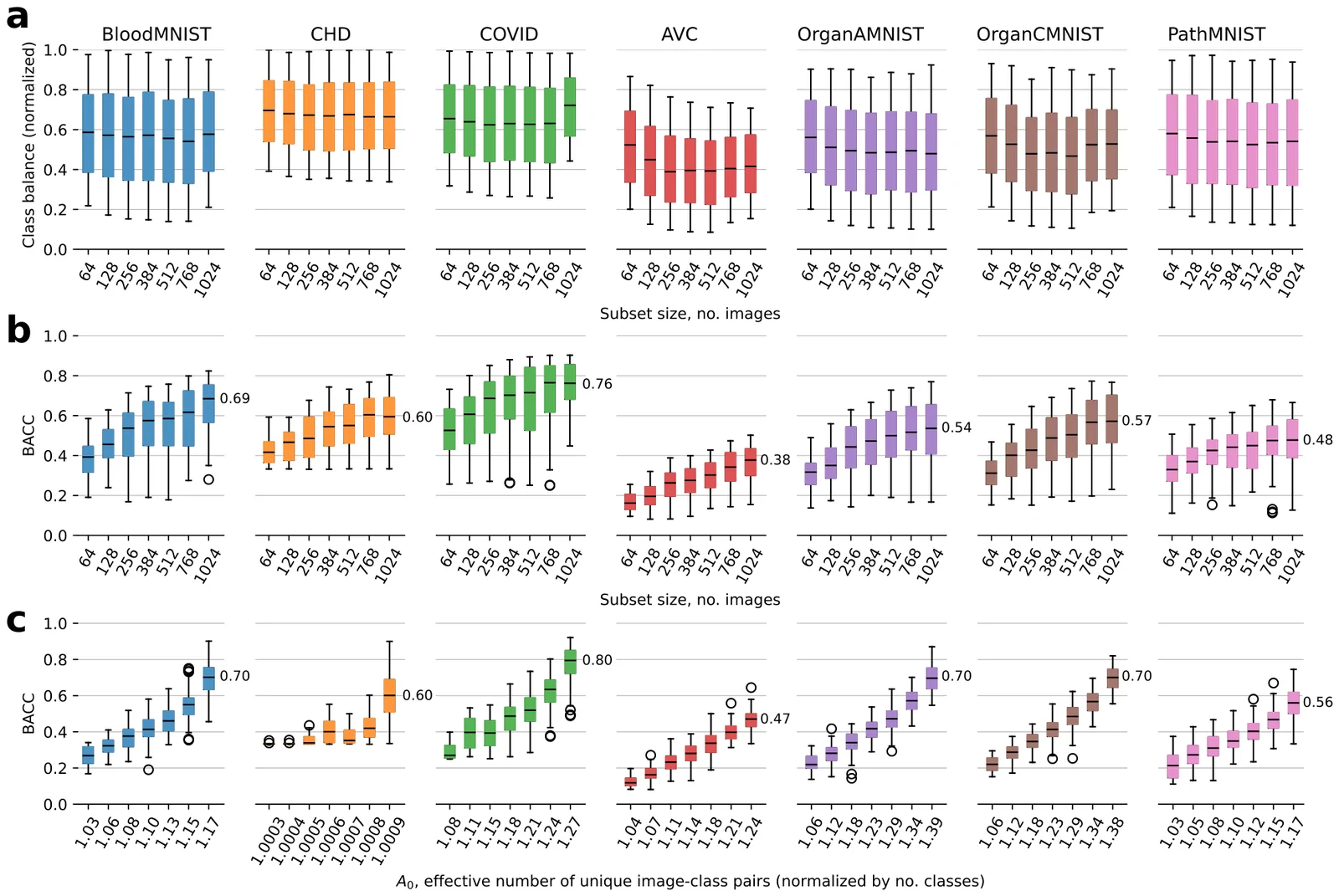
(**a**) Class balance vs. subset size, (**b**) BACC vs. subset size, and (**c**) BACC vs. $A_{0}$ (normalized by number of classes) for each dataset. Boxes span first and third quartiles; whiskers span an additional 1.5 interquartiles (the matplotlib.pyplot.boxplot default settings). Numbers in (**b**,**c**) indicate the median BACC for the top bin.

For each dataset, we created hundreds of subsets ranging from 64 to 1024 images in size that were designed to express the full range of class-balance values that are possible at each size (Figure 3a and Appendix A.7). The median number of subsets per dataset was 684 (range, 508–738). Note that creating class-balanced subsets from imbalanced datasets gets more difficult as subset size increases, since the number of images in the smallest class puts a limit on class balance; this was the rationale for 1024 as the maximum subset size. BACC spanned nearly the maximum possible range, from 0.08 to 0.92%, as desired; and rose with dataset size, as expected (Figure 3b).

### 4.4. Alpha Diversities as the Best Correlates of Model Performance/Dataset Quality

We used regression to assess how size, class balance, and diversity correlate with dataset quality. We tested 30 different features in all: 27 sentropies; two measures of class balance, namely the effective number forms of class Shannon entropy (${\mathrm{CB}}_{1}$) and class Berger–Parker index (${\mathrm{CB}}_{\infty }$); and subset size. Image size (no. pixels), color depth (no. channels), and the number of classes in the dataset were included in all regressions as normalizing factors, since these are constants within each dataset but can differ appreciably between datasets, and since they are unrelated to diversity.

#### 4.4.1. Best Single Correlates

**Figure 4:**
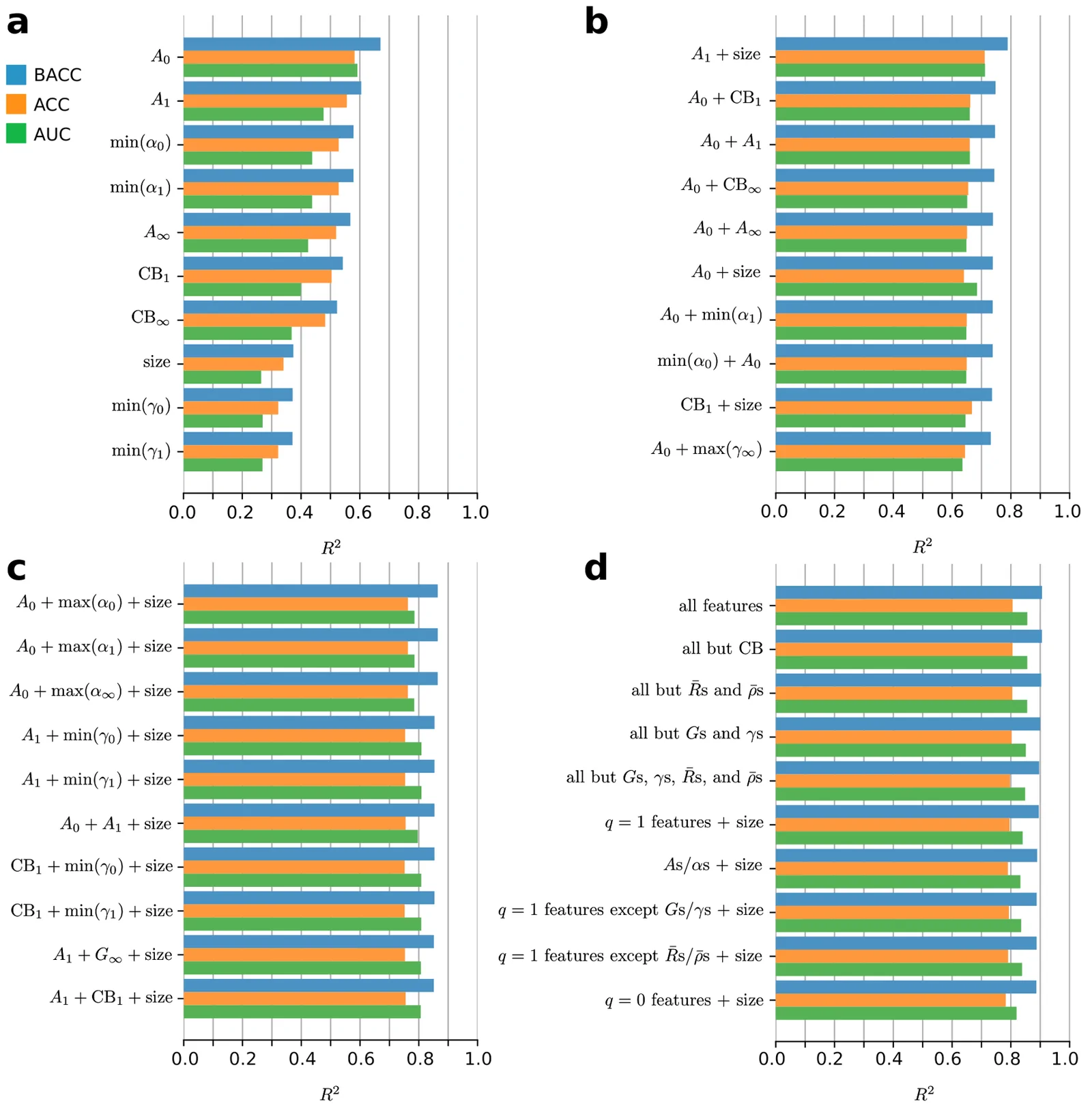
Regression results for (**a**) top 10 single measures, (**b**) top 10 pairs of measures, (**c**) top 10 sets of three measures, and (**d**) other sets of interest.

The five best individual correlates of dataset quality were all alpha diversities; using BACC as the indicator of performance, their mean $R^{2}$ was 0.600, meaning that on average they explained 60% of the variance in model performance (Figure 4a). $A_{0}$ or $A_{1}$ were also the best single correlate for each of the individual datasets, substantially better than CB in 5 of the 7 cases (Table 3). In contrast, CBs explained only a little over half the variance in performance ($R^{2}=$ 0.542 for ${\mathrm{CB}}_{1}$ and 0.523 for ${\mathrm{CB}}_{\infty }$) and dataset size explained only a little over a third ($R^{2}=$ 0.373). The best single correlate was $A_{0}$, which explained two-thirds of the variance in model performance ($R^{2}=$ 0.670). The second-best was $A_{1}$, which explained 60.5% ($R^{2}=$ 0.605). At the other extreme, the worst $R^{2}$ was 0.230 (for max $\gamma_{\infty }$). The pattern for ACC and AUC was similar but with systematically somewhat smaller values (Figure 4a).

$A_{0}$ was a visibly better correlate of BACC than size (Figure 3c vs. Figure 3b). Size exhibited plateauing/diminishing returns, as also seen in prior studies such as [33] (Figure 3b). Specifically, subsets with the largest $A_{0}$ were associated with a median of 8% higher performance than those with the largest size (range, 1–16%). The largest performance improvements were in the CT, AVC, and histopathology datasets (Figure 3b,c). The smallest effects on the medians were in BloodMNIST and CHD, but even in these datasets, the interquartile ranges reflected higher performances for $A_{0}$ than for size (Figure 3b,c; compare rightmost colored bars).

#### 4.4.2. Best Pairs of Correlates

The best pair of correlates across all datasets was $A_{1}$ plus dataset size, which together had an $R^{2}$ for BACC of 0.789 (Figure 4b). Nine of the 10 best pairs contained at least one alpha diversity measure; only three contained a CB and only three contained size. The pair of $A_{0}$ plus $A_{1}$ was third best at $R^{2}=$ 0.746, essentially tied with the second-place pair of $A_{1}$ plus ${\mathrm{CB}}_{1}$ ($R^{2}=$ 0.748). $A_{0}$ plus $A_{\infty }$, the only appearance of $A_{\infty }$ in the top 10 pairs, was fourth at $R^{2}=$ 0.739. The pair of size plus ${\mathrm{CB}}_{1}$ was ninth at $R^{2}=$ 0.74. For individual datasets, a pair containing an *A* measure was either best or second to CB plus size (Table 3).

#### 4.4.3. Other Groups of Correlates

**Figure 5:**
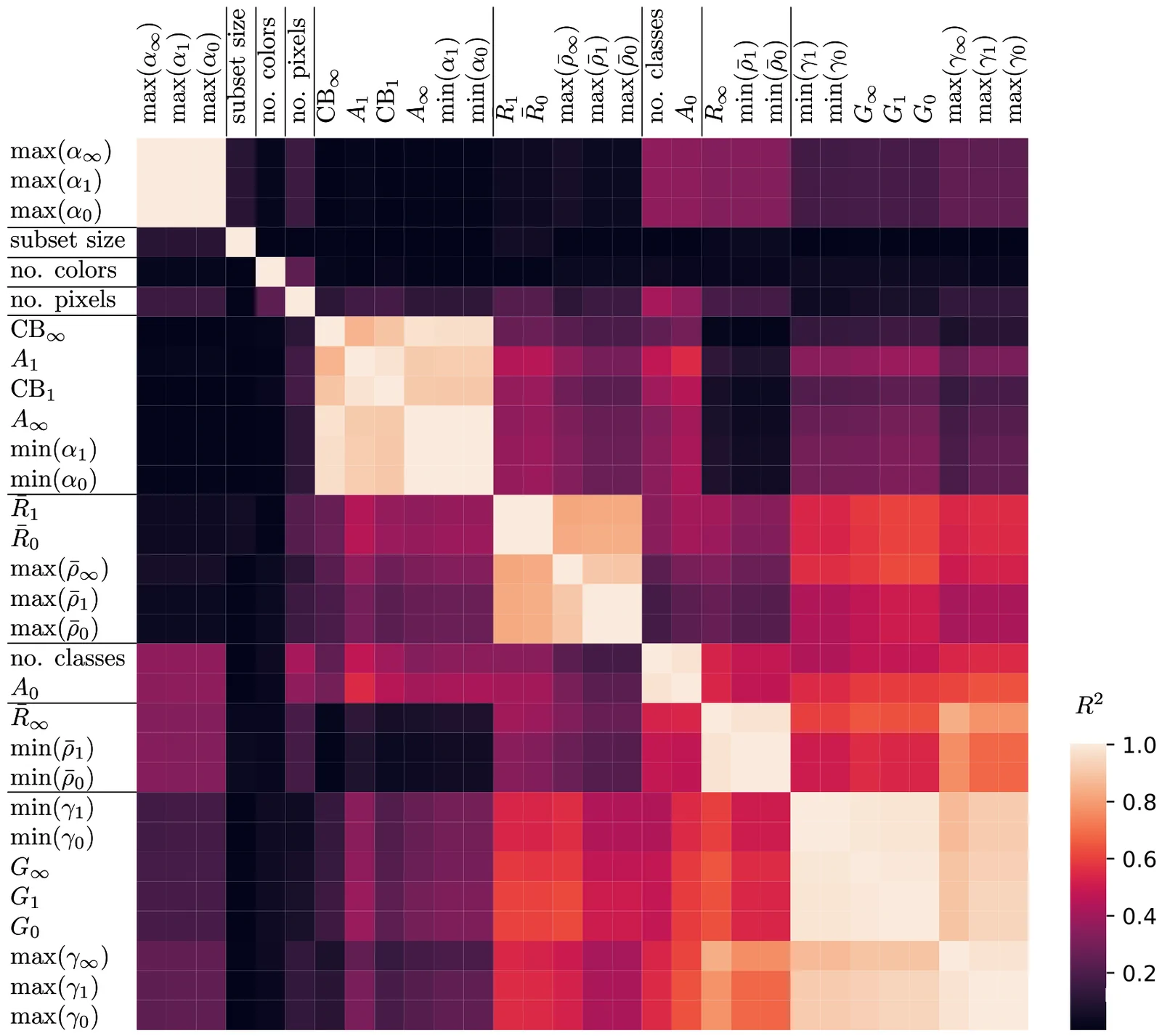
$R^{2}$ among sentropies (clustered). Lines delimit highly correlated clusters.

The top 10 groups of three correlates all performed similarly, with $R^{2}$ values of 0.840–0.851 for BACC (Figure 4c,d). Each included size, and eight of the 10 included $A_{0}$ or $A_{1}$. The only two groups that did not contain an *A* included ${\mathrm{CB}}_{1}$ and either min $\gamma_{0}$ or min $\gamma_{1}$. Pairwise correlations among the features are shown in Figure 5. Collectively, the eight alpha diversities tested—$A_{0}$, $A_{1}$, $A_{\infty }$, min $\alpha_{0}$, min $\alpha_{1}$, max $\alpha_{0}$, max $\alpha_{1}$, and max $\alpha_{\infty }$—explained 78.6% of the variance in performance. This was appreciably better than the eight gamma or rho diversities (62.4% and 59.0%, respectively) or the class balances (54.5%; almost identical to the 54.2% for ${\mathrm{CB}}_{1}$ by itself). For further comparison, recall from above that size on its own explained just 37.3%. Finally, the combination of all 27 diversity-related features, including size and class balance, explained 90.6% of the variance in model performance ($R^{2}=$ 0.906). Figure 4d shows additional feature sets of interest.

#### 4.4.4. Correlations Among Sentropies

High correlations were observed between many pairs of sentropies (Figure 5). These included between $A_{0}$ and $A_{1}$, between these *A*s and *G*s, and between these *A*s and the CBs.

## 5. Discussion

Although the primary approach to maximizing dataset diversity in imaging datasets, as an indicator or guarantor of quality, is to maximize dataset size and class balance, neither of these measures accounts for the diversity of the images themselves (Figure 1). In the present work, we show that sentropic dataset measures that account for the similarities and differences among images outperform size and class balance as indicators of dataset quality (at least as single measures, and pooled across our datasets), potentially offering ways to improve model performance without additional compute power or architectural improvements, by optimizing for *A* and/or other sentropies (which include size and class balance as special cases).

We find that the alpha-diversity sentropies $A_{0}$ and $A_{1}$ are better correlates of model performance than size or class balance, and are thereby better measures of dataset quality. Specifically, $A_{0}$ explained about two-thirds of the variance in BACC in the pooled case, or nearly twice as much variance as dataset size. Meanwhile, ${\mathrm{CB}}_{1}$ explained a little over half the variance. On average, datasets with the largest $A_{0}$ had a median 8% higher accuracy than those with the largest size (Figure 3b,c). We consider these meaningful improvements.

### 5.1. $A_{0}$ as the Top Correlate of Dataset Quality

Conceptually, $A_{q}$ is the effective number of unique image–class pairs in the dataset, down-weighting less-frequent pairs proportional to the given *q*, given the similarities and differences among the images. They measure the alpha diversity of a dataset: they are averages of the alpha diversities of the labeled classes (taking the similarities and differences among images into account). $A_{0}$ is simply the arithmetic mean of the $\alpha_{0}$ diversities of the classes; $A_{1}$ is reminiscent of Shannon entropy in that it down-weights the contributions of less diverse classes by an amount given by q=1.

We found that $A_{0}$, the best correlate of model performance, appreciably outperformed $A_{1}$ on the pooled data: a 6.5% absolute and 10.7% relative improvement (for BACC). This largely held in the single dataset regressions, though with one case where $A_{1}$ came out ahead. However, adding size reversed the relationship: $A_{1}$ plus size outperformed $A_{0}$ plus size, albeit by a smaller amount (5.1% absolute/6.9% relative improvement), consistent with $A_{1}$ being more independent of size. Interestingly, size and class balance together tied or beat pairs containing *A* in four of seven cases. We note that $A_{0}$ and $A_{1}$ were highly correlated with each other, and to class balance. It may be that *A* captures information in both size and class balance, making it more informative than either, but that both together capture similar information. Because we did not explicitly investigate methods for maximizing the different sentropies, it is not clear from the present work whether one would have to choose one or the other *A* to maximize, or whether both of them (and/or other sentropies) could be maximized simultaneously. Similarly, it is not obvious when it might be desirable to maximize one diversity measure at the expense of another. These are questions for future work.

### 5.2. Alphas vs. Other Sentropies

It is interesting that $A_{0}$ and $A_{1}$ both outperform *G* by a considerable margin. Indeed $A_{0}$ outperforms all the gamma-diversity measures combined (67.0% vs. 62.4% for all gammas combined). *G* differs from *A* in being the effective number of unique images in the dataset irrespective of class divisions. Thus, our results support the intuition that high-quality datasets should have diverse classes, not just diverse images. This is sensible: one would expect models to have an easier time learning determinative class invariants when each class represents diverse images. This understanding explains the much weaker performance of $A_{\infty }$ (which scored about as well as class balance): the q=∞ in $A_{\infty }$ means that this *A* focuses on just the single most diverse class. Our results show that the diversity of all classes matters, not just that of the most diverse class.

Of the dataset-level sentropies, the ones that showed the lowest correlation with performance were the $\bar{\rho }$s (28.3–35.0%) and *R*s (23.9–27.8%). These were absent from the top-correlating sets of features. Interestingly, together all $\bar{\rho }$s and *R*s explained 59.0% of the variance in BACC (better than CB). Recall that ${\bar{\rho }}_{q}$ measures how representative each class is of the overall dataset; as *q* increases, ${\bar{\rho }}_{q}$ focuses increasingly on the representativeness of the largest similarity block. $R_{q}$ is the power mean of ${\bar{\rho }}_{q}$ across the classes, interpreted as the amount of information gained about the class, by knowing the pixel values. (For example, for q=1, $-\log_{2}\bar{R}$ is the number of bits of information gained, which is ∼0 when $\bar{R}∼$ 1, and increases as $\bar{R}$ decreases.) The low correlations of individual $\bar{\rho }$s and *R*s indicate performance does not depend much on how representative the classes are of the dataset as a whole. Their much better performance as a set suggests some complementarity to each other. Exploring this further is left for future work.

The strong correlations observed among several sentropies are a consequence of the relationship $A=\mathrm{CB}\times G\times \bar{R}$ [42] (which holds precisely at q=1 and approximately, in practice, for many other *q*) and the generally high similarity between pairs of images according to the similarity measure we chose. The empirical observation that *A* correlates well with CB means that $G\times \bar{R}$ is fairly constant across the datasets in this study. Indeed, we observed that both *G* and $\bar{R}$ are fairly close to 1 (with *G* between 1 and 1.6). Recall that *G* is the effective number of unique images; it being close to 1 means the effective number of images in these datasets is always nearly 1, again a consequence of the scale of the similarity measure used in this study. Meanwhile, $\bar{R}$ being close to 1 indicates that knowing the pixel values adds little information regarding the class. This could be because of the large number of potential pixel distributions, or the relatively small number of classes in some of the datasets.

### 5.3. Limitations and Future Directions

To our knowledge, the present work is the first systematic, quantitative demonstration of the relative value of size, class balance, and other sentropies (a.k.a. similarity-sensitive Hill numbers or effective-number forms of similarity-sensitive Rènyi entropies) in machine learning, demonstrating these measures combined explain over 90% of the variance in BACC. However, there are several limitations and questions to be addressed in future work, in addition to those mentioned in the foregoing discussion.

First, we have considered the quality of the training set but not the validation and/or test sets. Prior work suggests that performance will be higher the more representative the validation and test sets are of the training set, but this is yet to investigate sentropically. Second, it will be interesting to explore the effect of different image-similarity measures. Here we tested a simple RMSD-based measure. A study considering other measures, e.g., from trained embeddings, would add to the robustness of the current work. Third and similarly, we considered only a single model architecture (ResNet-18). It will be interesting to test other models, including neural nets with different set of inductive biases, such as vision transformers, and classical non-neural models. Fourth, in order to facilitate normalization by image size, color depth, and number of classes, we used features’ logarithms for our regressor. Whether other potential transforms might be more informative or more natural remains to be seen; a different transform might account for the ∼10% of variance unaccounted for by the subset of sentropies we investigated. Fifth, it will be interesting to test how well *A* can predict which of two datasets will result in a higher-performing model, other things equal (such as size and class balance), as a potential aid to dataset creation/curation. Sixth, while the focus here was on medical-image classification, it will be interesting to test how our results generalize to other imaging datasets, to tasks other than classification (such as segmentation), and to non-imaging datasets (e.g., text, tabular data). Finally, we computed sentropies comprehensively ($O(n^{2})$ with dataset size *n*). The computational complexity can perhaps be reduced by an approximation scheme, such as a sparsification in which only similarity to each point’s *k*-nearest neighbors (for some *k*) is computed. This algorithmic exploration is left for future work.

Overall, the introduction of the rich framework of sentropy to machine learning is expected to open fruitful directions of future study.

## Figures and Tables

**Table 1:** Datasets used in this study.

Dataset	Image Size	No. Colors	Target	Modality	No. Classes	No. Train	No. Test
Path MNIST	28×28	3	Colon histopathology	Microscopy	9	89,996	7180
Blood MNIST	28×28	3	Blood smears	Microscopy	8	11,959	3421
OrganA MNIST	28×28	1	Abdomen	CT, axial	11	34,581	17,778
OrganC MNIST	28×28	1	Abdomen	CT, coronal	11	13,000	8268
COVID	299×299	1	Chest	X-ray	4	12,288	4096
CHD	80×80	1	Fetal ECG	Ultrasound	3	52,619	8773
AVC	80×60	1	Adult ECG	Ultrasound	15	179,612	23,096

**Table 2:** AUC, Accuracy (ACC), and Balanced Accuracy (BACC) of benchmark models for each dataset.

Data Set	AUC	ACC	BACC
PathMNIST	0.96	0.84	0.80
AVC	0.95	0.87	0.85
OrganCMNIST	0.99	0.89	0.88
CHD	0.98	0.92	0.88
OrganAMNIST	0.99	0.92	0.91
BloodMNIST	0.99	0.93	0.92
COVID	0.99	0.95	0.95

**Table 3:** Individual dataset results (best results in bold).

Features	Path	Blood	OrganA	OrganC	CHD	COVID	AVC
$A_{0}$	**0.664**	**0.793**	**0.852**	**0.869**	0.419	**0.707**	**0.789**
$A_{1}$	0.533	0.593	0.543	0.551	**0.578**	0.703	0.371
$CB_{1}$	0.528	0.584	0.511	0.514	0.578	0.703	0.340
$A_{0}$ + size	0.667	0.811	**0.874**	**0.892**	0.502	0.714	0.862
$A_{1}$ + size	0.709	0.837	0.854	0.857	0.879	0.826	**0.879**
$CB_{1}$ + size	**0.711**	**0.837**	0.849	0.850	**0.879**	**0.833**	0.876

## Data Availability

Datasets are available from https://medmnist.com/ (accessed on 21 July 2026). The sentropy Python package is available via PyPi at https://pypi.org/project/sentropy/ (accessed on 21 July 2026) or GitHub at https://github.com/ArnaoutLab/sentropy (accessed on 21 July 2026).

## Abbreviations

The following abbreviations are used in this manuscript:

ACC	Accuracy
AUC	Area under the (ROC) curve
BACC	Balanced accuracy
ROC	Receiver-operator characteristic

## Appendix A

### Appendix A.1. Dataset Diversity as a Function of Element Frequencies and Similarities

Motivating our approach is the observation that measures of dataset diversity make intuitive sense only if they account for both the relative frequencies of the elements in a dataset and the similarities and differences among these elements (Figure 1) [49].

The effect of relative frequencies is less important in the context of machine learning datasets because elements each generally appear only once (although consecutive video frames can be quite similar [54]), but is worth illustrating because it factors into the sentropy framework we use, as the relative sizes of overlapping similarity clusters (below). Consider two datasets that each contain five unique images (Figure 1a). In the first dataset, one image appears six times and the other four each appear only once, while in the second dataset, each image appears twice. Both datasets consist of 10 images, but the second dataset is intuitively more diverse, since the first is dominated by, and therefore to a first approximation consists of, just a single image.

To illustrate the effect of similarities and differences, consider another two datasets of 10 images, all unique, where the images in the first dataset are very similar to each other and those in the second dataset are very different from each other (Figure 1b). Even though the datasets are identical by every purely frequency-based measure—including size, Shannon entropy, and every Rényi entropy—the second dataset is intuitively more diverse. Thus, measures of dataset diversity must also incorporate similarity, in addition to frequency, to align with the intuitive sense of what “diversity” should mean.

### Appendix A.2. Hill’s D-Number Framework and Element Frequencies

Sentropy is based on Hill’s diversity indices [46], also known as the Hill numbers or D numbers:

(A1) $$\begin{array}{ccc} D^{q}\left(p\right) & = & {\left[\sum_{i}p_{i}^{q}\right]}^{\frac{1}{1-q}} \end{array}$$

(A2) $$\begin{array}{ccc} & = & \exp\left[H^{\alpha }\left(p\right)\right] \end{array}$$

$D^{q}\left(p\right)$ are the “(similarity-insensitive) diversities of order *q*.” They are the exponentials of the Rényi entropies $H^{\alpha }\left(p\right)$ [10] (α=q) and the reciprocals of the generalized (power) means of order q−1. *p* is a vector that represents the relative frequencies of unique elements such that $p_{i}$ represents the relative frequency of the $i^{th}$ element and $\sum_{i}p_{i}=1$.

The parameter *q* down-weights the contribution of rarer elements to the total. Notably, several common statistics are simple transformations of the Hill numbers at different *q*, with size, Shannon entropy, Simpson’s index, and the Berger–Parker index corresponding to q=0, 1, 2, and *∞*, respectively [48]. It is useful to think of these measures as an ordered set that differ purely by *q*, i.e., as counts that increasingly ignore rarer elements. D-number forms have the advantages of self-consistent partitioning, of using the same units regardless of *q*, and of having a common interpretation as giving the “effective number” of elements in the dataset, discounting rarer elements proportional to *q* [48]. Note that while Hill numbers account for the relative frequencies of elements in a dataset, they do not account for elements’ similarities.

### Appendix A.3. Sentropy and Element Similarities

Sentropy [40,41,42] generalizes Hill’s framework by accounting for similarity:

(A3) $$\begin{array}{ccc} D_{Z}^{q}\left(p\right) & = & {\left[\sum_{i}p_{i}{\left({\mathrm{Zp}}_{i}\right)}^{q-1}\right]}^{\frac{1}{1-q}} \end{array}$$

(A4) $$\begin{array}{ccc} & = & \exp\left[H_{Z}^{\alpha }\left(p\right)\right] \end{array}$$

$D_{Z}^{q}\left(p\right)$ is the similarity-sensitive diversity of order *q*. $H_{Z}^{\alpha }\left(p\right)$ is the corresponding similarity-sensitive Rényi entropy. The new piece relative to the Hill numbers is *Z*, the similarity matrix, whose entries $Z_{ij}$ represent the pairwise similarity between species *i* and species *j*. ${\mathrm{Zp}}_{i}=\sum_{j}Z_{ij}p_{j}$ is interpreted as how “ordinary” the $i^{\mathrm{th}}$ unique element is, given all the other elements in the dataset; the more “ordinariness” in the dataset, the less diverse it is [40]. Similarities $Z_{ij}$ are generally assumed to lie in the interval [0,1], with every species having a similarity of 1 with itself. It is often also sensible to assume that *Z* is symmetric ($Z_{ij}=Z_{ji}$). We follow these two assumptions in the present work.

### Appendix A.4. Dataset Size and Class Balance as Special Cases of Sentropy

Several quantities of interest emerge as special cases of sentropy. Dataset size is the special case of sentropy with Z=I (the identity matrix) and q=0. More generally, the Hill framework is the special case of sentropy with Z=I for any *q*, making the traditional/similarity-insensitive versions of Shannon entropy, Simpson’s index, and the Berger–Parker index also special cases of sentropy. By extension, $D_{Z}^{q}$ for Z≠I can be interpreted as the extended/generalized/similarity-sensitive versions of (the effective-number forms of) Shannon entropy, Simpson’s index, and so on, with the details of the similarity sensitivity depending on the choice of *Z*. In particular, when *Z* has a block structure in which $Z_{ij}=1$ when elements *i* and *j* are in the same labeled class and $Z_{ij}=0$ otherwise, $D_{Z}^{q}$ gives the class balance. For example, the Shannon entropy of the relative class sizes, in effective-number form, is $D_{Z}^{1}$: the effective number of classes in the dataset, accounting for their relative sizes. Thus, sentropy provides a logical organizing principle for a very wide range of measures of dataset diversity, including the ones used most often in machine learning.

### Appendix A.5. Class-Level and Dataset-Level Diversities

In sentropy, each labeled class of a dataset can be assigned an α, β, and γ diversity. These are each defined at a given *q*; for readability we include the subscript only when necessary. α, β, and γ correspond roughly to the diversity within the class, the diversity of the elements in the class relative to the overall dataset, and the diversity of the class as a whole relative to the overall dataset, respectively [42]. β diversity can be thought of as how distinct a class is, relative to the dataset as a whole. The corresponding quantities for the dataset as a whole are the power means across the classes and are denoted *A*, *B*, and *G* respectively (“big alpha,” “big beta,” and “big gamma”). The reciprocal of β, ρ=1/β, also has a useful interpretation as how representative a class is of the dataset as a whole; *R* is the corresponding dataset-wide measure, the power mean of ρ over all classes. Usefully, in sentropy, within- and between-class diversities are independent of each other [41,42]. (The same is true in the special case that is the Hill framework [48].) These properties are unique to sentropy [42].

As a terminological note, “datasets” and “unique elements” are examples of what Leinster, Cobbold, and Reeve’s original description of sentropy and the broader D-number/ecological literature refer to as “communities” and “species”, respectively; here we use whichever synonym is clearest, given the context.

### Appendix A.6. Interpretation

*Z* can be interpreted as describing a soft clustering of images based on their pairwise similarities (Figure 2). In this example, $Z_{ij}=e^{-{\mathrm{RMSD}}_{ij}}$ for images *i* and *j* was calculated for a nearly perfectly class-balanced but otherwise random subset of 128 images from the COVID dataset and clustered using the clustermap method of the seaborn Python package.

The heatmap in Figure 2 illustrates multiple soft, overlapping, or fuzzy clusters (lighter regions) across the four classes in this small dataset, which are represented by shaded bars along the top and left. ${\mathrm{CB}}_{1}$ is the class balance in effective-number form at q=1, i.e., the exponential of the class-balance Shannon entropy, a common way of expressing class balance in the literature. $A_{1}$ is the effective-number form of the power mean of similarity-sensitive Shannon entropies across the four classes, also at q=1 (the Shannon analogue). It is interpreted as the effective number of unique image–class pairs in the dataset, after accounting for similarity. The difference between class balance (an effective number of 3.96 classes, meaning all four classes are almost evenly represented) and $A_{1}$ (4.97 images, meaning this dataset has the same diversity as a dataset with just ~5 completely distinct/zero-pairwise-similarity images) can be interpreted as the diversity within the four classes, which class balance does not capture. The diversity $D_{Z}^{q}\left(p\right)$ of the dataset can then be thought of as the effective number of these clusters, with *Z* accounting for overlap and *q* describing how to weight the count based on the clusters’ relative sizes.

The ability for $Z_{ij}$ to take multiple/continuous values is what allows for soft/overlapping/fuzzy cluster membership. Which *q* or *q*s are of interest to the investigator depends on the context, although q=0 and q=∞ are natural bounds, with q=1 as a sensible midpoint; here we are primarily interested in investigating *q* that result in α, ρ, and γ and *A*, *R*, and *G* that correlate with the performance of deep-learning models on image-classification tasks for medical-image datasets.

### Appendix A.7. Data

Table A1 lists the number of subsets of each size selected from each parent dataset.

**Table A1:** Number of subsets of each size chosen from each dataset.

Dataset Name	64	128	256	384	512	768	1024	2048	Total
Path MNIST	88	93	96	96	93	94	96	50	706
Blood MNIST	90	95	97	100	96	96	88	40	702
OrganA MNIST	80	86	89	86	88	88	86	44	641
OrganC MNIST	81	88	84	87	85	79	80	30	614
CHD	92	96	100	100	100	100	100	50	738
COVID	92	96	100	100	100	100	76	20	684
AVC	73	74	71	70	69	65	61	25	508
total	596	628	637	639	631	622	587	259	4599
